# microRNA-mediated resistance to hypoglycemia in the HepG2 human hepatoma cell line

**DOI:** 10.1186/s12885-016-2762-7

**Published:** 2016-09-15

**Authors:** Satomi Ueki, Yuko Murakami, Shoji Yamada, Masaki Kimura, Yoshimasa Saito, Hidetsugu Saito

**Affiliations:** Department of Pharmacotherapeutics, Faculty of Pharmacy, Keio University, Minato-ku, Tokyo, 1058512 Japan

**Keywords:** Hepatoma, Hypoglycemia, MicroRNA, Resistance

## Abstract

**Background:**

It is generally accepted that the energy resources of cancer cells rely on anaerobic metabolism or the glycolytic system, even if they have sufficient oxygen. This is known as the Warburg effect. The cells skillfully survive under hypoglycemic conditions when their circumstances change, which probably at least partly involves microRNA (miRNA)-mediated regulation.

**Methods:**

To determine how cancer cells exploit miRNA-mediated epigenetic mechanisms to survive in hypoglycemic conditions, we used DNA microarray analysis to comprehensively and simultaneously compare the expression of miRNAs and mRNAs in the HepG2 human hepatoma cell line and in cultured normal human hepatocytes.

**Results:**

The hypoglycemic condition decreased the expression of *miRNA-17-5p* and *-20a-5p* in hepatoma cells and consequently upregulated the expression of their target gene *p21*. These regulations were also confirmed by using antisense inhibitors of these miRNAs. In addition to this change, the hypoglycemic condition led to upregulated expression of heat shock proteins and increased resistance to caspase-3-induced apoptosis. However, we could not identify miRNA-mediated regulations, despite using comprehensive detection. Several interesting genes were also found to be upregulated in the hypoglycemic condition by the microarray analysis, probably because of responding to this cellular stress.

**Conclusion:**

These results suggest that cancer cells skillfully survive in hypoglycemic conditions, which frequently occur in malignancies, and that some of the gene regulation of this process is manipulated by miRNAs.

**Electronic supplementary material:**

The online version of this article (doi:10.1186/s12885-016-2762-7) contains supplementary material, which is available to authorized users.

## Background

The most common cause of death in Japan has been cancer since 1981, with half of Japanese people suffering from cancer in their lifetime and one-third dying of cancer in recent years (http://www.mhlw.go.jp/english/database/db-hw/vs01.html). One reason for cancer development is genomic mutations. Another reason is epigenetic alterations that occur due to exposure to certain lifestyle factors, such as unhealthy foods, smoking, and drinking, or certain environmental factors, such as ultraviolet light and air pollution. Unfortunately, we still have no definite solution for cancer. Therefore, it is necessary to identify new therapeutic procedures by defining and exploiting the specific characteristics of cancers. One specific cancer target might lie in their metabolism.

The energy source of cells, including malignant cells, is adenosine triphosphate (ATP). ATP is mainly produced during the metabolism of glucose. Glucose metabolism involves glycolysis, the tricarboxylic cycle, and oxidative phosphorylation. The first two events occur in the cytoplasm, whereas the last event takes place in mitochondria. In hypoxic conditions, the reaction always stops at the stage of glycolysis. In 1921, it was reported that the oxygen consumption of cancer tissues was higher than that of normal tissues [1]. Accordingly, researchers have since focused on the abnormal metabolic state of cancer cells. Cancer cells cannot obtain enough oxygen and nutrition because they proliferate so fast that their nascent vasculature cannot keep up. This hypoxic condition facilitates the activation of a transcription factor, hypoxia-induced factor-1, and the transcription of glucose transporter and genes related to the glycolytic system [2].

On the other hand, there have been observations that the energy resources of cancer cells always depend on anaerobic metabolism or the glycolytic system, even if they have sufficient oxygen [3], namely, the Warburg effect, with both glycolysis and cell proliferation increasing in parallel [4]. These findings suggest a scenario whereby an abnormal state of the glycolytic system may be one of the mechanisms of carcinogenesis rather than a scenario in which hypoxia makes cancer cells adopt a hyperglycolytic condition. The glycolytic system has low energy-producing efficiency and only two ATP molecules are produced from one glucose molecule, in contrast to the 36 ATP molecules produced through oxidative phosphorylation. This means that more glucose is required for ATP production in the glycolytic system. However, as mentioned above, cancer cells always live under hypoglycemic conditions in which they themselves can obtain low levels of energy, and this condition appears to be contradictive. There are several explanations for this contradiction. One is that cancer cells can produce nucleic acids and amino acids necessary for their proliferation from an energy source other than ATP [1, 5]. Another is that cancer cells would like to avoid apoptosis without obtaining energy through mitochondria [6]. A final explanation is that cancer cells have a tolerance for glucose insufficiency. Cancer cells may skillfully live and proliferate in their hypoglycemic and hypoxic conditions.

Epigenetic alterations during carcinogenesis have received a great deal of attention. Recent investigations have revealed an important regulatory role for microRNA (miRNA) in epigenetic alterations in carcinogenesis and malignant transformation. miRNAs are 21–23 base-paired non-coding RNA that inhibit protein transcription by binding to target mRNA. One-third of all human protein-encoding genes is regulated by miRNA. In the present study, we focused on changes in the miRNA expression of liver cancer cells according to glucose concentration to investigate whether cancer cells adapt to altered glucose conditions by altering mRNA expressions.

## Methods

### Cells and culture conditions

The HepG2 cell line was used as a cancer cell line and was obtained from the American Type Culture Collection (Manassas, VA). In some of the experiments, long-term primary cultured human hepatocytes (HepaRG®, HEP220-MW24; Biopredic International, Saint Grégoire, France) were used as normal human hepatocytes [7]. HepG2 cells were cultured as described previously [8] in Dulbecco’s modified Eagle’s medium (DMEM; including 1000 mg/L of glucose) supplemented with 10 % fetal bovine serum (FBS). The human hepatocyte culture was maintained in long-term culture medium (MIL238-110 M) according to the conditions recommended by the manufacturer. Cells were cultured in a humidified incubator with 5 % CO_2_ at 37 °C.

We established three glycemic conditions. The hypoglycemic condition (200 mg/L glucose) comprised 20 mL DMEM (with 1000 mg/L glucose), 70 mL DMEM (without glucose), and 10 mL FBS per 100 mL. The normoglycemic condition (900 mg/L glucose) comprised 90 mL DMEM (with 1000 mg/L glucose) and 10 mL FBS per 100 mL. The hyperglycemic condition (1800 mg/L glucose) comprised 40 mL DMEM (with 4500 mg/L glucose), 50 mL DMEM (without glucose), and 10 mL FBS per 100 mL. The cells were cultured in 10-cm dishes (AGC Techno Glass Co., Ltd., Tokyo, Japan).

### Cell proliferation assay

Cell proliferation was assayed using a CellTiter 96® AQueous One Solution Cell Proliferation Assay (Promega KK, Tokyo, Japan). Cells were cultured in 96-well plates with 100 μL culture medium per well. Twenty microliters of CellTiter 96® AQueous One Solution Reagent was added to each well and the plates were incubated in a 5 % CO_2_ humidified incubator at 37 °C for 1 h. The absorbance at 490 nm was then measured with a microplate reader. The background was measured using culture medium alone without cells.

### Protein extraction and western blotting

Proteins were purified according to the method described previously [8] and their concentrations were measured with a BCA™ Protein Assay kit (Pierce Biotechnology Inc.). They were stored at −80 °C until use.

Proteins were separated on NuPAGE® 4–12 % Bis-Tris Gels (Life Technologies Japan Ltd., Tokyo, Japan) and transferred to iBlot™ Gel Transfer Stacks Nitrocellulose, Mini using iBlot™ (Life Technologies Japan Ltd.). The membranes were washed with PBS-T (0.01 M phosphate-buffered saline [PBS], 0.1 % Tween 20) and blocked with blocking buffer (0.01 M PBS, 0.02 % Tween 20, 5 % blocking agent) on a shaker at room temperature for 1 h. After washing with PBS-T, the membranes were incubated at 4 °C overnight with the following primary antibodies diluted in PBS-T (0.1 % Tween 20): anti-HSPA1B (ADI-SPA-810-D; 1:2000; StressGen), anti-HSPA8 (sc-7298; 1:500; Santa Cruz Biotechnology, Inc.), anti-p21 (sc-397; 1:5000; Santa Cruz Biotechnology, Inc.), anti-c-Myc (sc-40; 1:500; Santa Cruz Biotechnology, Inc.), or anti-actin (AC-40; 1:1000; Abcam). The membranes were then washed with PBS-T and incubated at room temperature for 1 h in secondary antibodies diluted in PBS-T (0.1 % Tween 20). After washing with PBS-T, the membranes were reacted with ECL Select Western blotting detection reagent (GE Healthcare UK Ltd., Little Chalfont, Buckinghamshire, UK) and fluorescence was detected.

### RNA extraction and DNA microarray

RNAs (including miRNAs) were extracted using the *mir*Vana™ miRNA Isolation Kit (Life Technologies Japan Ltd.) according to the recommendations of the manufacturer. Microarray analysis was carried out using the Human Oligo chip 25 K (ID QH0ZG35) by Toray Co. (http://www.3d-gene.com/en/about/; Tokyo, Japan) as described previously [9]. In brief, total RNA was amplified using an Amino Allyl aRNA kit (Ambion). RNA from cells was labeled with Cy3 Mono-Reactive Dye (GE Healthcare Japan Co., Tokyo, Japan), and control RNA was labeled with Cy5 Mono-Reactive Dye (GE Healthcare). After purification, each 1-μg sample was mixed and hybridized at 37 °C for 16 h. After washing, the hybridized chip was scanned using a 3D-Gene Scanner 3000 (Toray Co.). Background was subtracted from the raw data and the values were normalized according to a median Cy3/Cy5 ratio of 1.

### Reverse transcription

Reverse transcription for miRNA was done with a TaqMan® MicroRNA Reverse Transcription Kit (Applied Biosystems). Two nanograms/microliter of RNA was added to 0.15 μL of 100 mM dNTPs (with dTTP), 1.00 μL of MultiScribe™ Reverse Transcriptase (50 U/μL) (ThermoFisher Scientific K. K., Kanagawa, Japan), 1.50 μL of 10 × Reverse Transcription Buffer, 0.19 μL of RNase inhibitor (20 U/μL), and 3.0 μL of RT primer; distilled water (dH_2_O) was added to make 10 μL. Reverse transcription was performed using a Master Cycler Gradient (Eppendorf) at 16 °C for 30 s, 42 °C for 30 min, and 85 °C for 5 s and the product was stored at 4 °C. Reverse transcription for mRNA was performed according to the previously described method [10].

### Real-time polymerase chain reaction

The quantitative comparison of polymerase chain reaction (qPCR) products was performed by real-time PCR. qPCR of miRNA (reaction mixture: 10.0 μL of 2 × Universal PCR Master Mix II [Applied Biosystems], 7.0 μL of distilled water, 1.0 μL of TaqMan MicroRNA assay, and 2.0 μL of reverse transcription product) was done with a CFX96™ Real-Time System (Bio-Rad) (at 95 °C for 10 min, followed by 45 cycles at 95 °C for 15 s and 60 °C for 1 min). qPCR of mRNA was performed according to the method described previously [10]. Primers used were as follows: HSPA1B, F-TGGACTGTTG GGACTCAAGG AC, R-GGAACGAAAC ACCCTTACAG TATCA; HSPA8, F-TGCTGCTGCT ATTGCTTACG, R-TCAATAGTGA GGATTGACAC ATCA; p21, F-GACTCTCAGG GTCGAAAACG, R-GGATTAGGGC TTCCTCTTGG; and GAPDH, F-CACCACCATG GAGAAGGC, R-GCTAAGCAGT TGGTGGTGCA.

### Measurement of caspase-3 activity

Caspase-3 activity was measured with ApoAlert® Caspase Colorimetric Assay Kits (TaKaRa Clontech). Cell lysates from 2 × 10^6^ cells were centrifuged at 13,000 rpm at 4 °C for 10 min and 50 μL of the supernatant was applied to a 96-well microplate. Then, 50 μL of 2 × Reaction buffer/Dtt Mix and 5 μL of caspase substrate (N-acetyl-Asp-Glu-Val-Asp p-nitroanilide; DEVD-pNA) (TaKaRa Clontech) were added and the samples were incubated at 37 °C for 1 h. A standard curve was created and the absorbance of the sample was measured at 405 nm. The ratios of absorbance with and without H_2_O_2_ were calculated.

### Cell cycle analysis

The cells were fixed with 100 % ethanol at −20 °C overnight and reacted with 0.25 ng/mL of RNaseA at 37 °C for 1 h. The cells were then tagged with propidium iodide at room temperature for 30 min and analyzed with a flow cytometer, as described previously [8].

### Gene transfection

The *hsa-miR-17-5p mir*Vana® miRNA inhibitor (Ambion®, ThermoFisher Scientific K. K.) and *hsa-miR-20a-5p mir*Vana® miRNA inhibitor (Ambion®) were used to inhibit the expression of miRNAs. They were mixed with Lipofectamine® 3000 Reagent (ThermoFisher Scientific K. K.) and were incubated at room temperature for 5 min. The mixture was added to 70–90 % confluent HepG2 cells in 6-well culture dishes and the cells were incubated in 5 % CO_2_ at 37 °C for 48 h.

### Statistical analysis

Results are shown as the mean ± standard error (SE) and data were analyzed by one-way analysis of variance and multiple comparisons; the least significant difference method was used if the difference was significant. All comparisons are two-sided and a *p*-value <0.05 was considered significant. All statistical analyses were performed using SPSS 22 for Windows (SPSS, IBM Japan, Tokyo, Japan).

## Results

### The hypoglycemic but not hyperglycemic condition decreased the cell proliferation of hepatoma cells

**Fig. 1:**
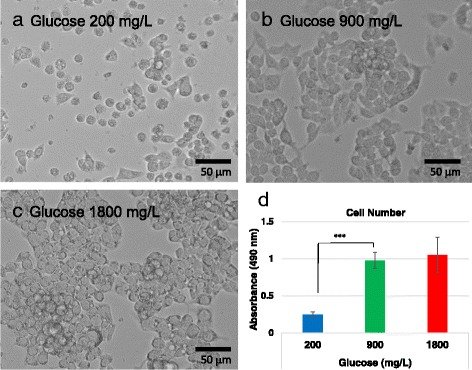
Cultured HepG2 cells after incubation with various concentrations of glucose. Representative microphotographs of HepG2 cells cultured with 200 (**a**), 900 (**b**), and 1800 (**c**) mg/L of glucose for 1 week (original magnification × 20). The results of the MTS assay are shown in (**d**). HepG2 cells were cultured with three different glucose concentrations (200, 900, and 1800 mg/L) for 1 week and cell number was measured with the MTS assay. There were fewer HepG2 cells when cells were cultured in the hypoglycemic condition (200 mg/L) than in the normoglycemic condition (900 mg/L). ***p < 0.001

HepG2 cells were cultured in medium containing normal glucose (900 mg/L), low glucose (200 mg/L), and high glucose (1800 mg/L) concentrations for 1 week, and viable cells were quantitatively assayed by the 3-(4,5-dimethylthiazol-2-yl)-5-(3-carboxymethoxyphenyl)-2- (4-sulfophenyl)-2H-tetrazolium, inner salt (MTS) assay. The numbers of viable cells decreased in low glucose conditions but were unchanged in high glucose conditions (Fig. 1).

### Microarray analyses showed several possible linkages between miRNA and mRNA expression

Comparative analyses of the expression of miRNAs (2555 genes) and mRNAs (24460 genes) of HepG2 cells and the human hepatocytes after incubation for 1 week in different culture conditions—high (1800 mg/L), normal (900 mg/L), and low (200 mg/L) glucose concentrations—were performed using 3D-Gene® (Toray Co.). In general, gene expression was more markedly changed in HepG2 cells than in normal HepaRG® human hepatocytes when cells were cultured under different glucose concentrations (Additional file 1: Figure S1). In the case of HepG2 cells, the pattern of genes that showed more than a 2-fold change under the hypoglycemic condition (200 mg/L) was as follows: 232 showing an miRNA increase, 37 showing an miRNA decrease, 1137 showing an mRNA increase, and 2078 showing an mRNA decrease. In the case of HepaRG® cells, the pattern of genes that showed more than a 2-fold change under the hypoglycemic condition was as follows: 13 showing a miRNA increase, 79 showing a miRNA decrease, 345 showing an mRNA increase, and 89 showing an mRNA decrease. Table 1 shows these significantly altered genes (the 10 genes showing the greatest increases and decreases in HepG2 cells and in HepaRG® cells) when the cell culture condition was changed from 900 mg/L to 200 mg/L glucose. Different genes were found to increase or decrease between HepG2 and HepaRG® cells.

**Table 1:** Microarray analysis results from HepG2 and HepaRG^®^ cells

miRNA	Glucose (mg/L)		Ratio	mRNA	Glucose (mg/L)		Ratio
	900	200			900	200	
A-1) The 10 genes showing the great increase in HepG2 cells							
*2003miR-150-3p*	35.2	185.4	5.27	*IFI27*	26	281	10.81
*miR-575*	97.3	471.9	4.85	*HSPA1B*	287	2009	7.00
*miR-3158-5p*	41.1	173.7	4.23	*IF16*	22	144	6.55
*miR-593-5p*	27.8	112.6	4.05	*CMBL*	53	296	5.58
*miR-1273e*	41.2	164.3	3.99	*SULF2*	19	106	5.58
*miR-6724-5p*	1080.5	4078.7	3.77	*CYP1A1*	25	135	5.40
*miR-671-5p*	71.8	265.8	3.70	*CAPN3*	54	286	5.30
*miR-4725-3p*	141.9	487.1	3.42	*DDB2*	125	624	4.99
*miR-8059*	154.7	520.5	3.36	*NT5DC4*	24	119	4.96
*miR-373-5p*	44.5	144.6	3.25	*LY96*	160	792	4.95
A-2) The 10 genes showing the great decrease in HepG2 cells							
*miR-106a-5p*	2174.7	1259.1	0.58	*KIF21A*	338	38	0.11
*miR-15b-5p*	243.3	148.8	0.61	*SLCC7A11*	273	33	0.12
*miR-93-5p*	684.5	421.1	0.62	*ANKRD12*	354	48	0.14
*miR-20a-5p*	1793.2	1219.7	0.68	*GOLGA4*	249	36	0.14
*miR-17-5p*	2144.4	1479.7	0.69	*TNKS2*	104	15	0.14
*miR-455-3p*	213.3	147.6	0.69	*MMD*	232	34	0.15
*miR-16-5p*	1133.7	792.8	0.70	*SLC38A2*	1313	202	0.15
*miR-19a-3p*	381.5	274.8	0.72	*ARHGAP18*	356	55	0.15
*miR-18a-5p*	306.7	220.9	0.72	*ZNF12*	116	19	0.16
*miR-26b-5p*	966.8	775.9	0.78	*ATP2B1*	244	40	0.16
B-1) The 10 genes showing the great increase in HepaRG^®^ cells							
*miR-6829-5p*	44.8	102.3	2.28	*RGS9*	129	280	2.17
*miR-3679-5p*	164.8	225.4	1.37	*SLC4A7*	113	226	2.00
*miR-4675*	177.4	234.8	1.32	*MY09A*	56	112	2.00
*miR-6726-5p*	87.3	104.7	1.20	*CAMSAP1L1*	62	123	1.98
*miR-6799-5p*	203.8	238.3	1.17	*SDCCAG1*	101	197	1.95
*miR-6803-5p*	737.2	818.5	1.11	*BCL9L*	250	483	1.93
*miR-6858-5p*	181.7	197.9	1.09	*PPHLN1*	266	497	1.87
*miR-6779-5p*	148.0	148.0	1.07	*BAT2L*	350	652	1.86
*miR-6721-5p*	150.4	150.4	1.06	*ZNF783*	77	142	1.84
*miR-6819-5p*	198.4	198.4	1.05	*RRAD*	74	136	1.84
B-2) The 10 genes showing the great decrease in HepaRG^®^ cells							
*miR-26b-5p*	205.5	85.9	0.42	*ESD*	252	124	0.49
*miR-191-5p*	102	44.5	0.44	*HMGCS1*	4950	2531	0.51
*let-7 g-5p*	212.4	101.1	0.48	*DDIT3*	205	109	0.53
*miR-19b-3p*	290.3	138.3	0.48	*MAP3K4*	107	57	0.53
*miR-20b-5p*	103.1	50.0	0.48	*GPR125*	121	66	0.55
*miR-1246*	4912.0	2388.6	0.49	*SMAD1*	101	55	0.54
*let-7e-5p*	215.6	107.8	0.50	*WBSCR1*	305	167	0.55
*miR-100-5p*	208.6	104.3	0.50	*PGM3*	132	72	0.55
*miR-148a-3p*	256.5	130.0	0.51	*THRSP*	442	253	0.57
*miR-122-3p*	215.8	112.9	0.52	*PSMB6*	1013	600	0.59

The result of microarray analysis of differently expressed miRNAs and mRNAs when HepG2 and HepaRG® cells were cultured in different glucose concentrations. Genes whose expression was greatly altered after the incubation of cells with two different concentrations of glucose, 900 mg/dL and 200 mg/dL, are shown. The 10 genes showing the great increase (A-1, B-1) and the 10 genes showing the greatest decrease (A-2, B-2) from 900 mg/L to 200 mg/L glucose are shown. The difference in the gene expression in HepG2 cells is shown in Table 1A, whereas that of HepaRG cells® is shown in Table 1B

When the cell culture condition was changed from normal (900 mg/L) to hyperglycemic (1800 mg/L), miRNA and mRNA expressions were also changed but the number of miRNAs and mRNAs affected was lower than that seen after the hypoglycemic change (Additional file 1: Figure S1). In HepG2 and HepaRG® cells, 79 and 9 miRNAs and 1011 and 335 mRNAs, respectively, showed a more than 2-fold increase in response to a change to the hyperglycemic condition (1800 mg/L). In HepG2 and HepaRG® cells, 15 and 81 miRNAs and 620 and 153 mRNAs, respectively, showed a more than 2-fold decrease in response to a change to the hyperglycemic condition. The 10 genes showing the greatest increase and the 10 genes showing the greatest decrease when the cell culture condition was changed from 900 mg/L to 1800 mg/L glucose concentration are shown in the Additional file 2: Table S1. The genes found to be increasing or decreasing were also different between HepG2 and HepaRG® cells, as for the change to the hypoglycemic condition.

We searched miRNA databases (http://mirdb.org/miRDB/ and http://www.targetscan.org) to identify expected gene regulators. In HepG2 cells under hypoglycemic conditions, we found a significant decrease in the expression of *miR-17-5p*, −*18a-5p*, −*19a-3p*, and *-20a-5p*, which together comprise the functional *miR-17/92* cluster (18). We found one candidate for a regulatory relationship between miRNA and mRNA expression, namely, heat shock protein A1B (HSPA1B), which is possibly regulated by *miR-15b-5p* and *miR-16-5p*. HSPA1B belongs to the 70-kDa heat shock protein (HSP70) family and is induced by some stresses such as heat or hypoxia to inhibit cellular apoptosis [11–13]. HSPA1B also plays a role as a chaperone, which keeps intracellular proteins in their normal state [14, 15]. On the other hand, in normal hepatocytes, *HSPA1B* expression was not significantly changed, although the expression of *miR-15b-5p* and *miR-16-5p* decreased under hypoglycemic conditions (Table 2). HSPA8 also belongs to the HSP70 family and inhibits apoptosis [12, 13], and this gene is targeted by *miR-17-5p* and *miR-20a-5p*, among the miRNAs shown in Table 1. Although expression of these two miRNAs greatly decreased (by about half), the expression of *HSPA8* was increased only about 1.17-fold in the hypoglycemic condition in HepG2 cells, and its expression was not changed in normal hepatocytes (Table 2). Given these results, we did not assess the changes in the expression of the mRNAs in the HepaRG® cells.

**Table 2:** The change in the expression of the possibly linked genes in HepG2 cells and normal HepaRG® hepatocytes detected by microarray analyses (200 mg/L glucose vs 900 mg/L)

miRNA	Glucose (mg/L)		Ratio	mRNA	Glucose (mg/L)		Ratio
	900	200			900	200	
a) HepaRG® (normal hepatocytes)							
*miR-15b-5p*	51	25.1	0.49	*HSPA1B*	139	140	1.01
*miR-16-5p*	325.5	209.2	0.64	*HSPA8*	5386	5148	0.96
*miR-17-5p*	162.9	96.8	0.59	*CDKN1A*	1439	1457	1.01
*miR-20a-5p*	122.7	76.5	0.62				
b) HepG2							
*miR-15b-5p*	243.3	148.8	0.61	*HSPA1B*	287	2009	7.00
*miR-16-5p*	1133.7	792.8	0.70	*HSPA8*	9925	11574	1.17
*miR-17-5p*	2144.4	1479.7	0.69	*CDKN1A*	291	713	2.45
*miR-20a-5p*	1793.2	1219.7	0.68				

The expression of *HSPA1B* mRNA was seen to be regulated by *miR-15b-5p* and *miR-16-5p*, and this regulatory relationship was observed in the results of microarray analyses in HepG2 cells; *HSPA1B* mRNA was not increased in HepaRG® cells. *HSPA8* transcription was not changed as much in either cell type. The expression of *CDKN1A* mRNA was seen to be regulated by *miR-17-5p* and *miR-20a-5p*, and this relationship was observed in HepG2 cells; *CDKN1A* mRNA transcription did not change in HepaRG® cells

*miR-17-5p* and *miR-20a-5p* decrease the expression of cyclin-dependent kinase inhibitor 1A (*CDKN1A*) [16], whose expression was increased about 2.45-fold in the hypoglycemic condition in HepG2 cells, although its expression was not changed in normal hepatocytes HepaRG® cells (Table 2) [16]. CDKN1A is the same as p21 and inhibits the G1/S checkpoint of the cell cycle, stopping cells in the G1 phase [17–19]. In response to these findings, we examined whether the expression of *HSPA1B* and *miR-15b-5p/16-5p* and that of *HSPA8/p21* and *miR-17-5p/20a-5p* were coupled.

As shown in Table 2, the baseline expression level of *HSPA1B* and *HSPA8* was much higher in HepG2 cells than in HepaRG® cells (287 vs. 139 and 9925 vs. 5386, respectively), suggesting that the baseline resistance to stresses might be much stronger in HepG2 cells than in HepaRG® cells. The baseline expression level of CDKN1A was much higher in HepaRG® cells than in HepG2 cells (1439 vs. 291), suggesting that there might be more S phase cells in HepaRG® cultures.

### Linkage between the expression of HSPA1B and the expression of miR-15b-5p and miR-16-5p

**Fig. 2:**
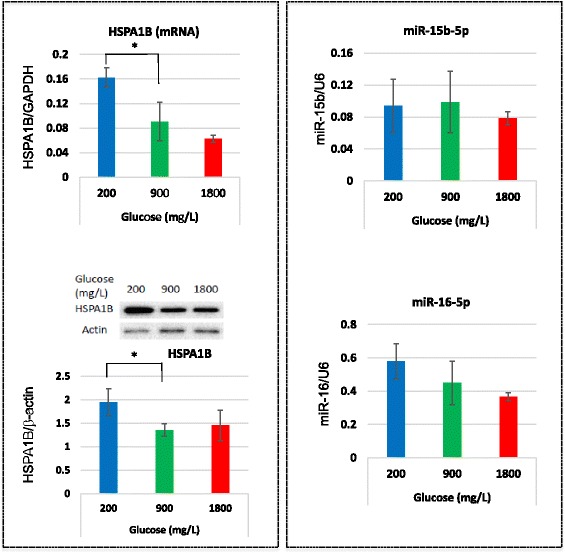
The expression levels of *miR-15b-5p* and −*16-5p* and their target gene HSPA1B in HepG2 cells after incubation with various concentrations of glucose. Cells were cultured with 200, 900, and 1800 mg/L of glucose for 1 week and qPCR and western blotting were performed to quantitate the expression of miRNAs and their target mRNA and protein. **a** The expression levels of mRNA and protein of HSPA1B. The graph shown under the western blotting images indicates the results of band densitometry analysis. **b** Expression levels of *miR-15b-5p* and *miR-16-5p*. Error bars indicate the standard error (SE) of four different experiments. In the case of western blotting, the SE was calculated from the densitometry data. *p < 0.05 (*n* = 4 each)

We confirmed the relationship between the expression of *miR15b-5p/16-5p* and HSPA1B. Both protein and mRNA expression levels of *HSPA1B* were found to increase in the low glucose condition using qPCR and western blotting (Fig. 2a), but the expression levels of *miR-15b-5p* and −*16-5p* were not changed (Fig. 2b). We could not confirm the microarray data in the low glucose condition in the case of *miR-15b-5p* and −*16-5p*.

### *Linkage between the target gene expression of the* miR-17/92 *cluster and c-Myc expression*

**Fig. 3:**
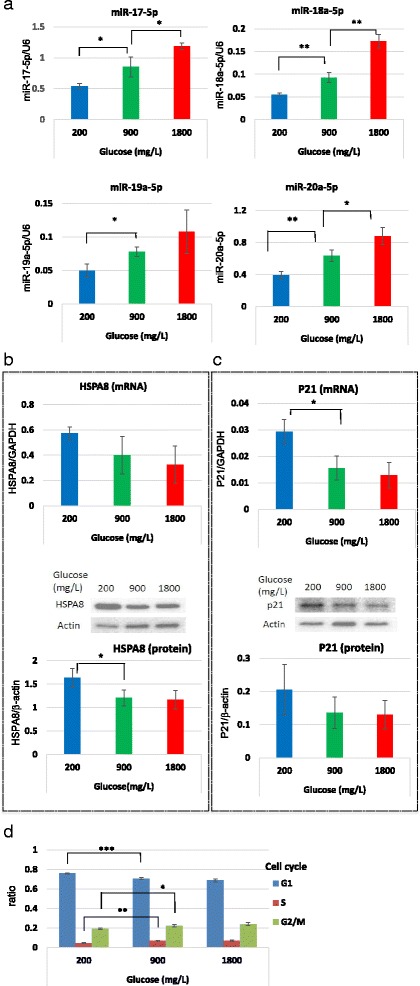
The expression levels of the miR-17/92 cluster and its target genes, HSPA1B and P21, in cells after incubation with various concentrations of glucose. **a** Cells were cultured with 200, 900, and 1800 mg/L of glucose for 1 week and the expression of the miR-17/92 cluster (*miR17-5p*, miR*-18a-5p*, miR*-19a-5p*, and miR*-20a-5p)* was analyzed. The expression levels of the target genes of the miR-17/92 cluster (HSPA8 and P21) were examined and the expression levels of mRNA (qPCR) and protein (western blotting) of HSPA8 (**b**) and P21 (**c**) were analyzed. The graph shown under the western blotting images indicates the band densitometry results. The cell cycle was analyzed with a flow cytometer after the cells were stained with propidium iodide (**d**). Error bars indicate the standard error (SE) of four different experiments. In the case of the western blotting, the SE was calculated from the densitometry data. *p < 0.05, **p < 0.01, ***p < 0.001 (*n* = 4 each)

Real-time PCR was performed to confirm the decrease in the expression of the *miR-17/92* cluster in the low glucose condition. The expression of *miR-17-5p*, −*18a-5p*, −*19a-3p*, and *-20a-5p* was significantly decreased in the low glucose condition and was significantly increased in the high glucose condition (Fig. 3a).

Because HSPA8 and p21 are reported to be targets of *miR-17/92* (http://mirdb.org/miRDB/ and http://www.targetscan.org), we examined their expressions in the low glucose condition, finding an increase in the mRNA and protein expressions of HSPA8 and p21 (Fig. 3b, c). We next examined whether the glucose concentration affects p21 expression by assessing the cell cycle with flow cytometry (Fig. 3d). The hypoglycemic condition increased p21 expression in HepG2 cells. In addition, the proportion of cells in the G1 phase significantly increased, whereas that of cells in the S and G2/M phases significantly decreased under the hypoglycemic condition. When cells were incubated under hyperglycemic conditions, no change was noticed in the cell cycle phase.

Because c-Myc facilitates transcription of the *miR-17/92* cluster, we examined c-Myc expression in the low glucose condition using western blotting. However, its expression was not altered (Additional file 3: Figure S2).

**Fig. 4:**
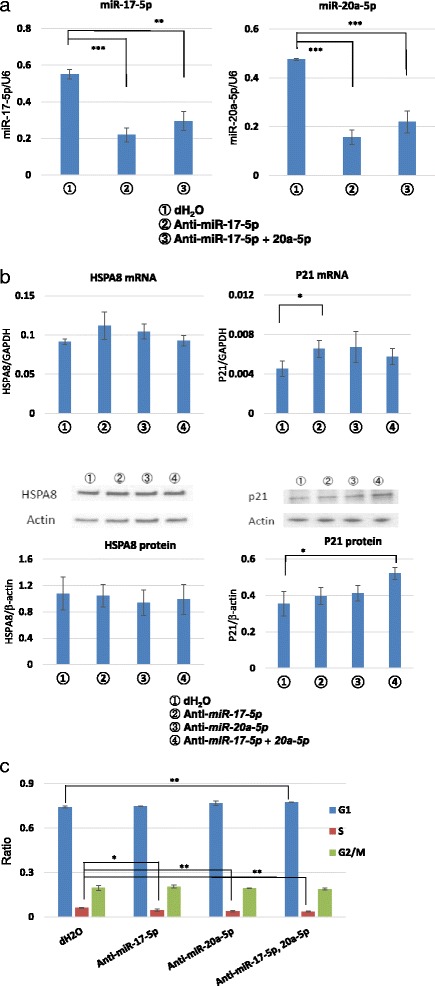
Effects of the *hsa-miR-17-5p* and *hsa-miR-20a-5p mir*Vana® miRNA inhibitors. **a** Effects of miRNA inhibitors indicated by ①-③ on the expression levels of *miR-17-5p* and *-20a-5p*. **b** Effects of miRNA inhibitors indicated by ①-④ on the expression of HDPA8 and p21. The expression of HDPA8 and P21 were examined by both qPCR and western blotting. The graph shown under the western blotting images indicates the band densitometry results. **c** Cell cycle analysis after the transfection of cells with miRNA inhibitors. The ratios of each cell cycle phase are shown as a bar graph. Blue color indicates G1 phase, red color indicates S phase, and green color indicates the G2/M phase cell ratio. Error bars indicate the standard error (SE) of four different experiments. In the case of western blotting, the SE was calculated from the densitometry data. *p < 0.05, **p < 0.01, ***p < 0.001 (*n* = 4 each)

We next transfected the antisense RNA for *miR-17-5* and *miR-20a-5p* into HepG2 cells cultured under the normoglycemic condition. The expression levels of *miR-17-5* and *miR-20a-5p* were significantly suppressed by transfection of the antisense inhibitors (Fig. 4a). However, the mRNA and protein expression levels of HSPA8 were not altered (Fig. 4b). On the other hand, the *miR-17-5p* inhibitor significantly increased the transcription of *p21* (Fig. 4b) and protein expression was significantly inhibited when both *miR-17-5* and *miR-20a-5p* were inhibited with the antisense RNA (Fig. 4b). The inhibition of both *miR-17-5* and *miR-20a-5p* increased the proportion of G1 phase cells and decreased the proportion of S phase cells (Fig. 4c).

### Increased expression of HSPA1B and HSPA8 in the hypoglycemic condition confers resistance to H_2_O_2_-induced apoptosis

**Fig. 5:**
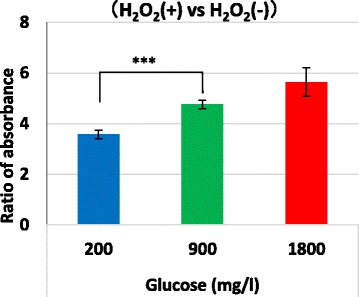
Caspase-3 activity induced by hydrogen peroxide in cells after incubation with various concentrations of glucose. Cells were cultured with 200, 900, and 1800 mg/L of glucose for 1 week and then 0.8 mM of hydrogen peroxide was added to the culture. After incubation for 24 h, cells were collected and caspase-3 activity was measured with absorbance at 405 nm. The ratios of the absorbance with and without H_2_O_2_ were calculated. Error bars indicate the standard error (SE) of four different experiments, as calculated from the data of the calculated ratios. ***p < 0.001 (*n* = 4 each)

The HSP family comprises anti-apoptotic factors that protect cells from various stresses by inhibiting caspase-3 activity. We examined whether cellular resistance to apoptosis was enhanced by the increased expression of HSPA1B and HSPA8 in the low glucose condition. We added H_2_O_2_ (final concentration, 0.8 mM) to induce oxidative stress in the last 24 h of a 7-day culture of HepG2 cells under the different glucose conditions. HepG2 cells cultured in the low glucose condition were resistant to apoptotic stress due to increased caspase-3 activity compared with cells cultured in the normal glucose condition. On the other hand, HepG2 cells cultured in the high glucose concentration seemed to show greater activation of caspase-3 (Fig. 5).

## Discussion

Cancer cells mainly depend on the glycolysis system for energy and have to adapt to environmental glucose conditions. Here, we found evidence of miRNA-mediated changes in human hepatoma-derived HepG2 cells in response to environmental glucose concentrations. Under a hypoglycemic condition, HepG2 cells became resistant to apoptosis by increasing HSP expression and regulated the cell cycle by changing p21 expression via miRNAs.

Because we wanted to examine whether some conditions occur only in cancer cells, we used HepaRG® cells as a control. As shown in Fig. 2, the change in the gene expression of HepG2 cells according to the glucose condition was much larger than that of normal hepatocytes. We cannot evaluate whether the normal hepatocytes we used worked well or not, but HepG2 cells were sensitive to the glucose concentration in their surroundings, which was reflected in the intracellular signaling. Fig. 1 shows that miRNA expression in HepG2 cells always increased and that mRNA expression generally decreased in the hypoglycemic condition. On the other hand, in general, both the hypo- and hyperglycemic conditions resulted in decreased miRNA expression in normal hepatocytes, which increased mRNA expression. Thus, cancer cells seemed more sensitive to glycemic conditions and the gene expression was different to, or in other words, the opposite of that of normal cells.

Among genes whose expression was upregulated in the hypoglycemic condition, HSPA1B inhibits cellular apoptosis by inhibiting cytochrome c release from mitochondria and downstream caspase-3 activation in HepG2 cells [13]. HSPA1B might play an important role in HepG2 cells because the expression of HSPA1B was maintained at relatively high levels in HepG2 cells, even in the normoglycemic condition, and its expression was not changed in normal hepatocytes. We confirmed the increased expression of this anti-apoptotic gene under the hypoglycemic condition, but the levels of the anticipated regulatory miRNAs, *miR-15b* and *miR-16,* did not change, and other miRNA regulatory factors were not found. Further investigation is necessary to clarify the mechanism of this regulation.

HSPA8, which is a member of the HSP70 family, also exerts an anti-apoptotic function [12, 13] by inhibiting caspase-3 activation [13]. We confirmed that HepG2 cells under the hypoglycemic condition were resistant to hydrogen peroxide-induced apoptosis, probably due to upregulation of HSPA1B and HSPA8. HSPA8 expression, as well as that of HSPA1B, was significantly increased in HepG2 cells under the hypoglycemic condition and this increase was not detected in normal hepatocytes. We investigated whether this increased expression was linked with decreased expression of *miR-17-5p* and *-20a-5p* by inhibiting these miRNAs with antisense RNA. However, inhibition of *miR-17-5p* and *-20a-5p* did not alter the HSPA8 expression, suggesting that there is no regulatory correlation between HSPA8 and *miR-17-5* and *-20a-5p.* Although we did not identify the miRNA-related regulatory mechanism of the upregulation of HSPA expression, a hypoglycemic condition seemed to render cancer cells resistant to apoptotic stimulation.

One of the target genes of *mir-17-5p* and *-20a-5p* is p21, which is a cell cycle regulator [16]. In HepG2 cells, the expression of p21 was increased in the hypoglycemic condition and knockdown of *mir-17-5p* and *-20a-5p* increased the expression of p21. The change in the expression of p21 was minor in normal hepatocytes, suggesting that cancer cells were more sensitive to the effect of low glucose on the increase in p21 expression. p21 inactivates the cyclin/cyclin-dependent kinase complex at the G1/S checkpoint in the cell cycle and induces G1/S arrest [17–19]. In HepG2 cells, the number of G1 phase cells was increased in the hypoglycemic condition while that of S and G2/M phase cells was decreased in response to an increased expression of p21. These changes in the cell cycle and p21 expression were also found in cells transfected with the inhibitors for *mir-17-5p* and *-20a-5p*. These results suggest that a decreased glucose supply induces the cell cycle arrest of HepG2 cells in response to decreased expression of *mir-17-5p* and *-20a-5p* and the subsequent p21 increase. This metabolic change in HepG2 cells may be an adaptive response of cancer cells to a limited glucose supply in which the cells reduce their energy consumption.

We found that changes in the glucose concentration induced the expression of *miR-17-5p*, −*18a*, −*19a*, and *-20a-5p* but we could not identify the factors regulating these miRNAs. We considered that these miRNAs were regulated as a cluster. One of the known regulatory factors of the *miR-17/9* cluster is c-Myc but we could not find any change in the expression of this protein. One possible explanation is that there was a change in the binding affinity between c-Myc and the *miR-17/92* cluster; another possible explanation involves the transcription factor E2F, which is reported to regulate the expression of the *miR-17/92* cluster [20]. Further studies are needed to identify the regulatory factors of the *miR-17/92* cluster that might link changes in the glucose concentration and miRNA expression.

We found using microarray analysis that interferon alpha-inducible protein 27 (IFI27) and IFI6 were upregulated in the hypoglycemic condition. These proteins are induced by interferon stimulation. The expression of IFI27 and IFI6 is upregulated in epithelial malignancies, breast cancer, and ovarian cancer [21–25], and their expression may be abundant in cancer cells. Interferon is a cytokine that protects cells from viral infection and the upregulated expression of IFI27 and IFI6 might be a protective response of cancer cells to certain stresses, as well as to the upregulation of HSP expression. Recently, Roulois et al. and Chiappinelli et al. showed that DNA-demethylating agents can induce a cell-autonomous immune active response by stimulating the expression of dsRNA-containing endogenous retroviruses [26, 27]. We have also demonstrated induction of an anti-viral response with the DNA methylation inhibitor 5-aza-2′-deoxycytidine in an intestinal tumor organoid [28]. Thus, it seems that biological stresses induce an anti-viral response and that this response might be epigenetically regulated by DNA demethylation. Another upregulated gene, lymphocyte antigen 96 (*LY96*), is also an important factor in innate immunity, involved in the binding between lipopolysaccharide and toll-like receptor 4, indicating that not only an anti-viral response, but also an innate general immunity might be activated. Changes in these immunological regulators triggered by a hypoglycemic environment suggest that an accommodation of cancer cells to the hypoglycemic condition allows them to resist attacks from unfavorable foreign enemies, and it was also possible that a hypoglycemic condition induced demethylation of some genes. Further investigation of the epigenetic changes of some specific genes may clarify these mechanisms. The other genes found in the microarray analysis, such as *CMBL* (a cysteine hydrolase of the dienelactone hydrolase family), *Sulf2* (a sulfatase), *CAPN3* (a calcium-activated neutral protease or a non-lysosomal intracellular cysteine protease), and *DDB2* (a DNA damage-binding protein), are enzymes with various functions. These genes might also help cancer cells to withstand several cellular stresses in the hypoglycemic condition.

In the present study, we investigated how cancer cells survive in the hypoglycemic condition, which frequently occurs in vivo in patients with malignancies, regardless of how cancer cells depend on glucose as an energy source. Upregulated expression of p21 via miRNA-mediated epigenetic mechanisms and HSPs without regulation by miRNAs occurred in HepG2 cells but not in normal cells under the hypoglycemic condition. Further investigation with in vivo studies is required to validate these phenomena.

## Conclusions

HepG2 cells, a liver cancer cell line, seemed to adapt themselves to the hypoglycemic condition, which always occurs in human malignancies, and one of the mechanisms of their adaptation involved miRNA-dependent regulation. Our findings showed that HSPs were upregulated by hypoglycemia in this cell line and that the cell cycle was at least partly arrested via miRNA-dependent p21 upregulation.

## Additional files

Additional file 1: Figure S1. DNA microarray analysis after cells were cultured with various concentrations of glucose. HepG2 and HepaRG® cells were cultured with glucose concentrations of 200, 900, and 1800 mg/L for 1 week and the RNA extracted was analyzed by DNA array (3D-Gene®, Toray; http://333.3d-gene.com/). Plots of mRNA expressions in HepG2 and HepaRG® cells cultured with 900 mg/L (vertical axis) and 200 mg/L (horizontal axis) glucose (a and c, respectively). MicroRNA expressions in HepG2 and HepaRG® cultured with 1800 mg/L (vertical axis) and 900 mg/L (horizontal axis) glucose are plotted in b and d, respectively. (PPTX 1966 kb) Additional file 2: Table S1. Change in the expression of the indicated genes between the hyperglycemic and normoglycemic conditions. Genes whose expression was greatly altered after the incubation of cells with two different concentrations of glucose, 900 mg/dL and 1800 mg/dL, are shown. The 10 genes showing the greatest increase and the 10 genes showing the greatest decrease from 900 mg/L to 1800 mg/L glucose are shown. The difference in the gene expression in HepG2 cells is shown in Table 1a, whereas that of HepaRG cells ® is shown in Table 1b. (DOCX 120 kb) Additional file 3: Figure S2. c-Myc expression after incubation of cells with various concentrations of glucose. Cells were cultured with 200, 900, and 1800 mg/L of glucose for 1 week and the expression of c-Myc protein was examined by Western blotting. The graph under Western blotting shows the result of densitometry of bands. No significant change in this gene expression was detected in different glucose concentrations. (PPTX 89 kb)

## Abbreviations

ATP: Adenosine triphosphate
CDKN1A: Cyclin-dependent kinase inhibitor 1A
DEVD-pNA: N-acetyl-Asp-Glu-Val-Asp p-nitroanilide
DMEM: Dulbecco’s modified Eagle’s medium
FBS: Fetal bovine serum
IFI27: Interferon alpha-inducible protein 27
LY96: Lymphocyte antigen 96
miRNA: microRNA
MTS: 3-(4,5-dimethylthiazol-2-yl)-5-(3-carboxymethoxyphenyl)-2-(4-sulfophenyl)-2H- tetrazolium, inner salt
PCR: Polymerase chain reaction
qPCR: Quantitative polymerase chain reaction
SE: Standard error
