# Supplementary material for: microRNA-mediated resistance to hypoglycemia in the HepG2 human hepatoma cell line
Source: BMC Cancer. 2016 Sep 15;16:732. doi: 10.1186/s12885-016-2762-7 (PMC5024426; doi:10.1186/s12885-016-2762-7)
Supplement: Additional file 1: Figure S1. — DNA microarray analysis after cells were cultured with various concentrations of glucose. HepG2 and HepaRG® cells were cultured with glucose concentrations of 200, 900, and 1800 mg/L for 1 week and the RNA extracted was analyzed by DNA array (3D-Gene®, Toray; http://333.3d-gene.com/). Plots of mRNA expressions in HepG2 and HepaRG® cells cultured with 900 mg/L (vertical axis) and 200 mg/L (horizontal axis) glucose (a and c, respectively). MicroRNA expressions in HepG2 and HepaRG® cultured with 1800 mg/L (vertical axis) and 900 mg/L (horizontal axis) glucose are plotted in b and d, respectively. (PPTX 1966 kb) [file 12885_2016_2762_MOESM1_ESM.pptx]

## Slide 1
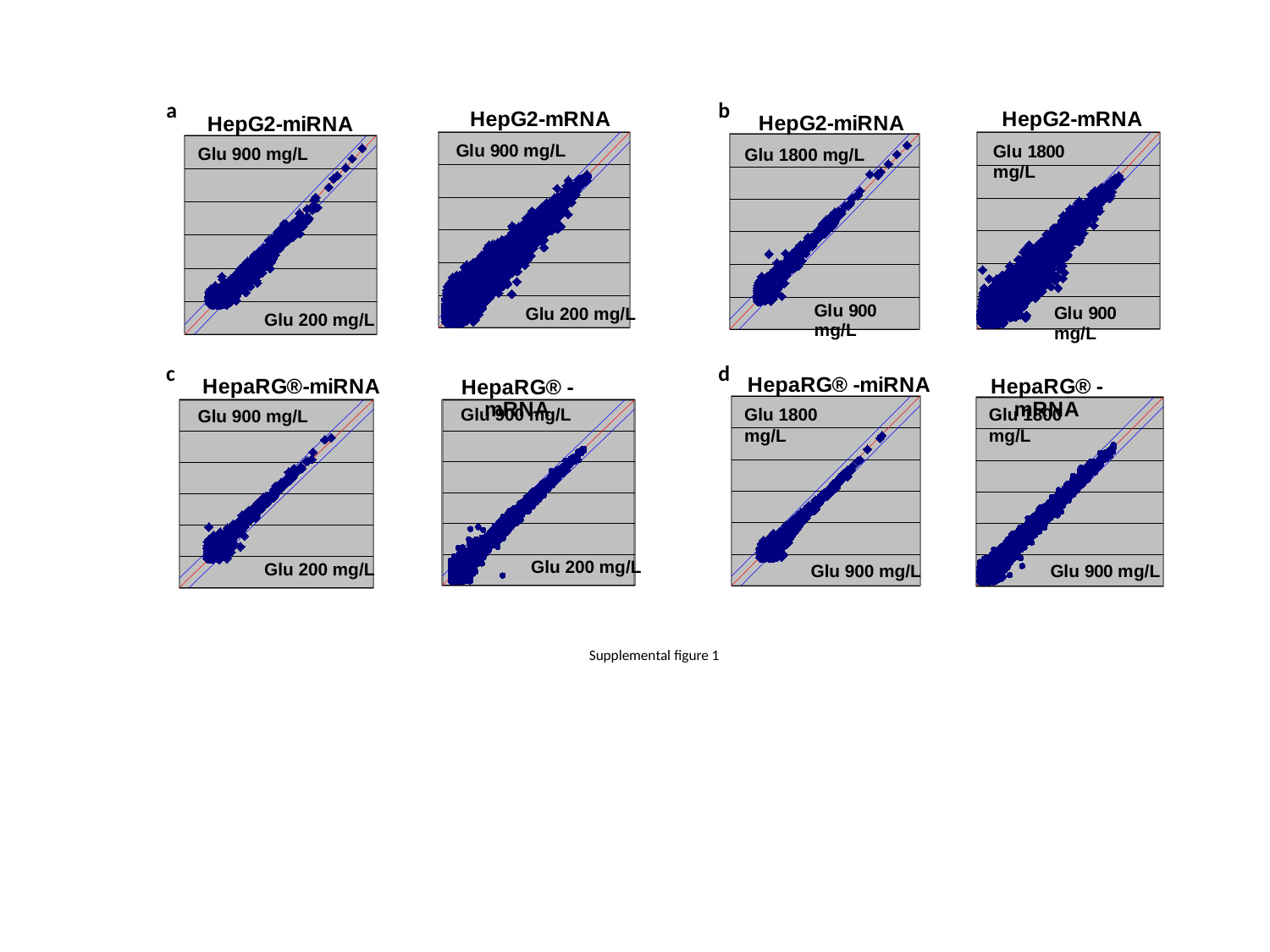

a
b
### Chart: HepG2-mRNA
| Category | | | | |
|---|---|---|---|---|
### Chart: HepG2-mRNA
| Category | | | | |
|---|---|---|---|---|
### Chart: HepG2-miRNA
| Category | | | | |
|---|---|---|---|---|
### Chart: HepG2-miRNA
| Category | | | | |
|---|---|---|---|---|Glu 900 mg/L
Glu 1800 mg/L
Glu 200 mg/L
Glu 200 mg/L
c
### Chart: HepaRG® -mRNA
| Category | | | | |
|---|---|---|---|---|d
### Chart: HepaRG® -miRNA
| Category | | | | |
|---|---|---|---|---|
### Chart: HepaRG® -mRNA
| Category | | | | |
|---|---|---|---|---|
### Chart: HepaRG®-miRNA
| Category | | | | |
|---|---|---|---|---|Glu 1800 mg/L
Glu 900 mg/L
Glu 1800 mg/L
Glu 900 mg/L
Glu 200 mg/L
Glu 200 mg/L
Glu 900 mg/L
Supplemental figure 1
