# Supplementary material for: microRNA-mediated resistance to hypoglycemia in the HepG2 human hepatoma cell line
Source: BMC Cancer. 2016 Sep 15;16:732. doi: 10.1186/s12885-016-2762-7 (PMC5024426; doi:10.1186/s12885-016-2762-7)
Supplement: Additional file 2: Table S1. — Change in the expression of the indicated genes between the hyperglycemic and normoglycemic conditions. Genes whose expression was greatly altered after the incubation of cells with two different concentrations of glucose, 900 mg/dL and 1800 mg/dL, are shown. The 10 genes showing the greatest increase and the 10 genes showing the greatest decrease from 900 mg/L to 1800 mg/L glucose are shown. The difference in the gene expression in HepG2 cells is shown in Table 1a, whereas that of HepaRG cells ® is shown in Table 1b. (DOCX 120 kb) [file 12885_2016_2762_MOESM2_ESM.docx]

**Supplemental Table: Change in the expression of the indicated genes between the hyperglycemic and normoglycemic conditions**

**a) HepG2 Glucose 900 mg/L vs 1800 mg/L**

| mRNA | Glucose (mg/L) | | Ratio |
| --- | --- | --- | --- |
|  | 900 | 1800 |  |
| *VGF* | 451 | 18 | 0.04 |
| *C13orf15* | 660 | 34 | 0.05 |
| *AQP3* | 740 | 52 | 0.07 |
| *SH2D5* | 267 | 19 | 0.07 |
| *EMP3* | 369 | 30 | 0.08 |
| *AQP3* | 408 | 37 | 0.09 |
| *NCF2* | 425 | 41 | 0.1 |
| *S100A2* | 135 | 14 | 0.1 |
| *KLF5* | 109 | 12 | 0.11 |
| *SLC17A3* | 166 | 20 | 0.12 |

**The decreased genes**

| miRNA | Glucose (mg/L) | | Ratio |
| --- | --- | --- | --- |
|  | 900 | 1800 |  |
| *miR-21-3p* | 150.7 | 86.4 | 0.57 |
| *miR-4465* | 196.7 | 126.7 | 0.64 |
| *miR-4485* | 166.5 | 112.2 | 0.67 |
| *miR-4747-3p* | 113.6 | 76.9 | 0.68 |
| *miR-1185-1-3p* | 100.5 | 72.2 | 0.72 |
| *miR-491-5p* | 179.8 | 131.3 | 0.73 |
| *miR-3178* | 1822.7 | 1497.1 | 0.82 |
| *miR-6724-5p* | 1080.5 | 896.5 | 0.83 |
| *miR-4690-3p* | 258.4 | 216.2 | 0.84 |
| *miR-3195* | 428 | 359.9 | 0.84 |

**The increased genes**

| miRNA | Glucose (mg/L) | | Ratio |
| --- | --- | --- | --- |
|  | 900 | 1800 |  |
| *miR-519e-5p* | 17.7 | 206.1 | 11.64 |
| *miR-3131* | 59.2 | 213.9 | 3.61 |
| *miR-708-5p* | 31.5 | 110.4 | 3.5 |
| *miR-130b-3p* | 71.3 | 162 | 2.27 |
| *miR-5191* | 172 | 384.9 | 2.24 |
| *miR-4496* | 302.6 | 673.9 | 2.23 |
| *miR-6976-5p* | 95.3 | 210.2 | 2.21 |
| *miR-422a* | 122.1 | 263 | 2.15 |
| *miR-4284* | 27365.2 | 57810.6 | 2.11 |
| *miR-338-3p* | 136.5 | 287.7 | 2.11 |

| mRNA | Glucose (mg/L) | | Ratio |
| --- | --- | --- | --- |
|  | 900 | 1800 |  |
| *PGC* | 51 | 364 | 7.14 |
| *SERPINA7* | 36 | 257 | 7.14 |
| *SERPINA11* | 33 | 222 | 6.73 |
| *HP* | 22 | 131 | 5.95 |
| *ASB9* | 22 | 129 | 5.86 |
| *CFI* | 57 | 324 | 5.68 |
| *MIXL1* | 25 | 121 | 4.84 |
| *C9orf103* | 24 | 118 | 4.92 |
| *CPB2* | 27 | 124 | 4.59 |
| *GNMT* | 49 | 225 | 4.59 |

**b) HepaRG^®^　Glucose 900 mg/L vs 1800 mg/L**

| mRNA | Glucose (mg/L) | | Ratio |
| --- | --- | --- | --- |
|  | 900 | 1800 |  |
| *GSDM* | 537 | 228 | 0.42 |
| *SDR39U1* | 406 | 176 | 0.43 |
| *CCL2* | 156 | 73 | 0.47 |
| *MAP3K4* | 107 | 54 | 0.5 |
| *HOXD9* | 433 | 232 | 0.54 |
| *IKBKE* | 500 | 272 | 0.54 |
| *AC020663.7* | 921 | 512 | 0.56 |
| *APBB2* | 162 | 94 | 0.58 |
| *BR11-129B22.1* | 938 | 547 | 0.58 |
| *RGS9* | 129 | 75 | 0.58 |

**The decreased genes**

| miRNA | Glucose (mg/L) | | Ratio |
| --- | --- | --- | --- |
|  | 900 | 1800 |  |
| *miR-197-5p* | 119.4 | 63.2 | 0.53 |
| *miR-3614-5p* | 100.1 | 54.4 | 0.54 |
| *miR-1290* | 422 | 232.6 | 0.55 |
| *miR-423-5p* | 110.3 | 61.4 | 0.56 |
| *miR-6879-5p* | 486.1 | 273.3 | 0.56 |
| *miR-575* | 103.6 | 58.5 | 0.56 |
| *miR-3185* | 135.2 | 76.7 | 0.57 |
| *miR-513a-5p* | 122.7 | 69.8 | 0.57 |
| *miR-4695-5p* | 187.3 | 107.7 | 0.58 |
| *miR-557* | 203.4 | 117.4 | 0.58 |

| mRNA | Glucose (mg/L) | | Ratio |
| --- | --- | --- | --- |
|  | 900 | 1800 |  |
| UGT1A1 | 46 | 143 | 3.11 |
| PDIA6 | 122 | 321 | 2.63 |
| CFH | 106 | 276 | 2.6 |
| THRSP | 442 | 1122 | 2.54 |
| PCSK9 | 99 | 250 | 2.53 |
| PTPLAD1 | 101 | 251 | 2.49 |
| HPR | 1284 | 3193 | 2.49 |
| UGT1A8 | 107 | 261 | 2.4 |
| APOB | 2347 | 5740 | 2.45 |
| CCPG1 | 109 | 266 | 2.44 |

**The increased genes**

| miRNA | Glucose (mg/L) | | Ratio |
| --- | --- | --- | --- |
|  | 900 | 1800 |  |
| *miR-612* | 84.7 | 113.5 | 1.34 |
| *miR-6860* | 123.4 | 161.2 | 1.31 |
| *miR-6790-5p* | 81.8 | 104.1 | 1.27 |
| *miR-6774-5p* | 159.6 | 201.1 | 1.26 |
| *miR-1247-3p* | 283.9 | 338.8 | 1.19 |
| *miR-6822-5p* | 204.9 | 234.5 | 1.14 |
| *miR-4687-5p* | 177 | 201.1 | 1.12 |
| *miR-30e-5p* | 141.8 | 158.4 | 1.12 |
| *miR-194-5p* | 462.3 | 507.9 | 1.1 |
| *miR-6782-5p* | 109.7 | 118.6 | 1.08 |

Genes whose expression was greatly altered after the incubation of cells with two different concentrations of glucose, 900 mg/dL and 1800 mg/dL, are shown. The 10 genes showing the greatest increase and the 10 genes showing the greatest decrease from 900 mg/L to 1800 mg/L glucose are shown. The difference in the gene expression in HepG2 cells is shown in Table 1a, whereas that of HepaRG cells ^®^ is shown in Table 1b.
