# Supplementary figures and images for: microRNA-mediated resistance to hypoglycemia in the HepG2 human hepatoma cell line
Source: BMC Cancer. 2016 Sep 15;16:732. doi: 10.1186/s12885-016-2762-7 (PMC5024426; doi:10.1186/s12885-016-2762-7)

## Slide 1
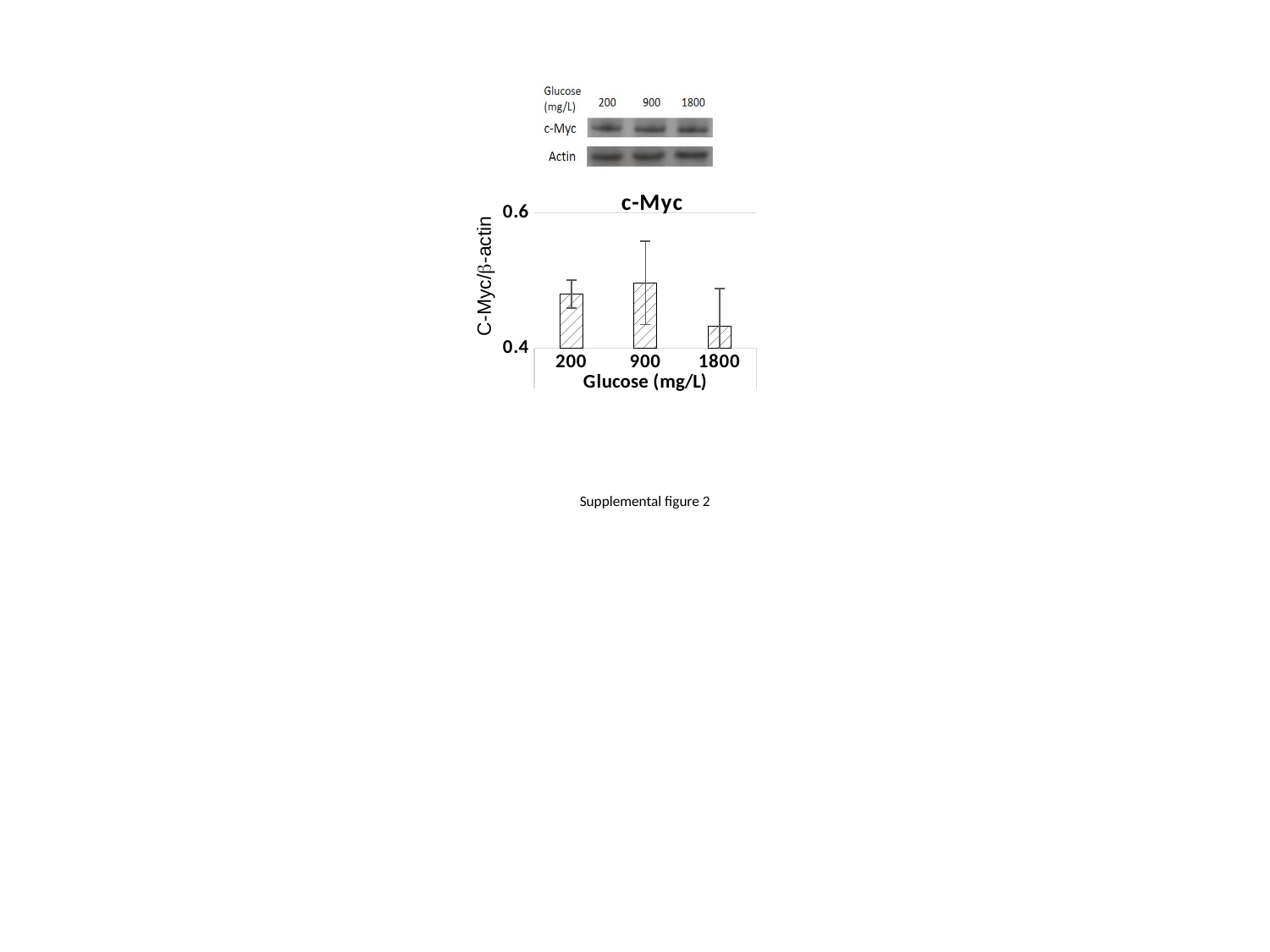

### Chart: c-Myc
| Category | |
|---|---|
| 200 | 0.479791126745895 |
| 900 | 0.496678622322788 |
| 1800 | 0.432715405473281 |C-Myc/b-actin
Supplemental figure 2

Supplement: Additional file 3: Figure S2. — c-Myc expression after incubation of cells with various concentrations of glucose. Cells were cultured with 200, 900, and 1800 mg/L of glucose for 1 week and the expression of c-Myc protein was examined by Western blotting. The graph under Western blotting shows the result of densitometry of bands. No significant change in this gene expression was detected in different glucose concentrations. (PPTX 89 kb) [file 12885_2016_2762_MOESM3_ESM.pptx]
